# Geminiviruses: a tale of a plasmid becoming a virus

**DOI:** 10.1186/1471-2148-9-112

**Published:** 2009-05-21

**Authors:** Mart Krupovic, Janne J Ravantti, Dennis H Bamford

**Affiliations:** 1 Department of Biological and Environmental Sciences and Institute of Biotechnology, Biocenter 2, P.O. Box 56 (Viikinkaari 5), FIN-00014 University of Helsinki, Helsinki, Finland

## Abstract

**Background:**

Geminiviruses (family *Geminiviridae*) are small single-stranded (ss) DNA viruses infecting plants. Their virion morphology is unique in the known viral world – two incomplete *T *= 1 icosahedra are joined together to form twinned particles. Geminiviruses utilize a rolling-circle mode to replicate their genomes. A limited sequence similarity between the three conserved motifs of the rolling-circle replication initiation proteins (RCR Reps) of geminiviruses and plasmids of Gram-positive bacteria allowed Koonin and Ilyina to propose that geminiviruses descend from bacterial replicons.

**Results:**

Phylogenetic and clustering analyses of various RCR Reps suggest that Rep proteins of geminiviruses share a most recent common ancestor with Reps encoded on plasmids of phytoplasmas, parasitic wall-less bacteria replicating both in plant and insect cells and therefore occupying a common ecological niche with geminiviruses. Capsid protein of *Satellite tobacco necrosis virus *was found to be the best template for homology-based structural modeling of the geminiviral capsid protein. Good stereochemical quality of the generated models indicates that the geminiviral capsid protein shares the same structural fold, the viral jelly-roll, with the vast majority of icosahedral plant-infecting ssRNA viruses.

**Conclusion:**

We propose a plasmid-to-virus transition scenario, where a phytoplasmal plasmid acquired a capsid-coding gene from a plant RNA virus to give rise to the ancestor of geminiviruses.

## Background

The origin(s) of viruses is a longstanding but yet unresolved question in biology. Several hypotheses were put forward in efforts to understand this enigma (reviewed in [1]). According to the "Virus-first" hypothesis, viruses emerged in the prebiotic world, just before or in parallel with cellular organisms [2,3]. The "Reduction" hypothesis states that viruses evolved by reduction from free-living ancient cellular lineages [4], while the alternative "Escape" hypothesis suggests that viruses originated from cellular genomic fragments that became free of their cellular environment [5]. Irrespective of which of the viral origin hypotheses is considered, these converge in the appreciation of the extreme antiquity of viruses, with origin(s) possibly predating the emergence of the last universal common ancestor (LUCA) of cellular organisms. The ancient origin of viruses is inferred not only from bioinformatic investigations [2] but, perhaps more convincingly, from the recent flow of structural information on a number of individual viral proteins as well as entire virions. Structural comparison of viruses infecting hosts from all three domains of life (Bacteria, Archaea, and Eukarya) revealed that certain viruses utilize very similar assembly principles and can be grouped accordingly into structure-based viral lineages [6,7]. The viral lineage hypothesis predicts that viruses existed at the time of (or even before) LUCA and their diversification into bacterial, archaeal and eukaryotic viruses was associated with the emergence of the three cellular domains. But do all virus families come from the dawn of life or can we still witness the more recent emergence of new viral families?

Plasmids comprise another group of parasitic genetic elements that inhabit cells in all three domains of life. Resemblance of plasmids to DNA viruses is apparent, especially when DNA replication strategies are considered [2]. Nevertheless, evolutionary relationships between these two groups are far from being understood. Obviously, the main (and in some cases the only) difference is the presence of the capsid protein-coding gene in the viral genome. For example, there are a number of cryptic plasmids that encode a single protein responsible for DNA replication, while some small viruses of the *Circoviridae *family bear only two genes [8,9], one for genome replication and the other one for capsid formation. Members of another virus family, *Nanoviridae*, contain multipartite genomes where each genomic segment contains a single gene and is packed into a separate isometric capsid [10]. For example, *Faba bean necrotic yellows virus *contains up to eleven chromosomes [11]. Of special interest are plant-infecting satellite RNA viruses, such as *Satellite tobacco necrosis virus *(STNV), that encode a single capsid protein and depend on helper viruses for genome replication. It is thus reasonable to assume that acquisition of a capsid gene by a plasmid or, vice versa, loss of a capsid gene by a virus will result in the transition from a plasmid to a genuine virus or from a virus to a plasmid, respectively. This hypothesis should be testable by scrupulous analysis of replication and capsid protein sequences and/or structures.

**Figure 1:**
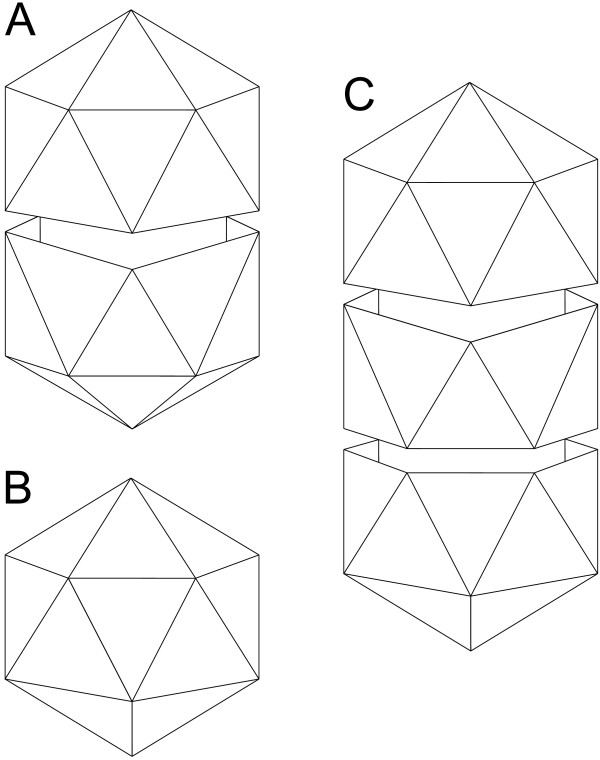
**Schematic representation of viral particles that can be built using capsid protein of geminiviruses**. (A) Wild-type twinned particle. (B) Isometric icosahedral particle. (C) Particle composed of three incomplete icosahedra. This representation is highly simplified for clarity; in reality the two icosahedra of the wild-type particle (A) are twisted to each other by 20° [33].

Geminiviruses (family *Geminiviridae*) are small insect vector-transmitted plant-infecting viruses. Their circular single-stranded (ss) DNA genome is encapsidated into twinned particles that are formed by joining two incomplete *T *= 1 icosahedra (Fig. 1A). According to the genome organization, host range and the insect vector used, geminiviruses are divided into four genera: *Mastrevirus*, *Curtovirus*, *Begomovirus *and *Topocuvirus*. Peculiarly, a number of begomoviruses possess bipartite genomes, i.e. genes are distributed on two separate ssDNA molecules that are usually both required for productive infection, while mastre-, curto- and topocuviruses encode all their genes on a single chromosome [12]. Geminiviruses replicate their genomes in the nuclei of infected (usually phloem tissue) cells via the rolling-circle (RCR) mechanism initiated by virus-encoded replication initiation protein (Rep) that ranges in size from approximately 320 to 400 amino acid residues. In addition to the three conserved motifs, typical to RCR Reps [13], geminiviral Reps possess a carboxy-terminal helicase domain with Walker A and B motifs [14,15]. The ATPase activity of the geminivirus Rep protein was proven to be essential for replication [16]. Koonin and Ilyina (1992) observed a limited sequence similarity between the three conserved motifs of the RCR Reps of geminiviruses and plasmids of Gram-positive bacteria and suggested that geminiviruses descend from bacterial replicons [17]. Here we tested this hypothesis by thoroughly analyzing a set of capsid and RCR protein sequences from geminiviruses.

## Results and Discussion

Geminiviruses are plant pathogens and due to their agricultural importance, a great number of sequences from geminiviral isolates has been determined and deposited into databases. We generated a specific sequence pattern to select from the non-redundant BLAST database (including environmental protein sequences) all 1072 protein sequences sharing conserved motifs with Rep proteins of geminiviruses. Many of these sequences are almost identical; therefore, in order to avoid redundancy, the initial dataset was filtered to leave only sequences that are less than 70% identical to each other. After subsequent manual examination, the final dataset contained 40 sequences (see Methods for data collection details). Nineteen of these belonged to geminiviruses, while the rest were from a marine metagenome project (6 sequences), circoviruses (6 sequences), phytoplasmal plasmids (5 sequences), plasmid of *Porphyra pulchra *(1 sequence), nanovirus (1 sequence), *Bifidobacterium catenulatum *DSM 16992 (1 sequence), and *Nicotiana tabacum *(1 sequence). Interestingly, the latter sequence was previously concluded to originate from integration of geminiviral DNA into the plant chromosome [18]. Nanoviruses and circoviruses are small icosahedral viruses with ssDNA genomes. While nanoviruses infect plants, circoviruses replicate in mammalian or avian cells. Bifidobacteria are gram-positive bacteria residing in the gastrointestinal tract of humans and other warm-blooded animals. Interestingly, Rep from *B. catenulatum *DSM 16992 is homologous to a Rep of the *Bifidobacterium pseudocatenulatum *plasmid p4M [GenBank:AAM00235], which has been previously observed to be similar to Reps of circoviruses [19]. Phytoplasmas are parasitic bacteria infecting the phloem tissue of plants. Phytoplasmas belong to the class of Mollicutes, which encompasses small pleiomorphic wall-less bacteria, also including mycoplasmas, ureaplasmas, spiroplasmas and acholeplasmas [20]. Phytoplasmas are transmitted by insects that feed on the phloem of infected plants [21,22]. It should be noted that geminivirus-related bacterial RCR Reps, other than those from phytoplasmal plasmids and *B. catenulatum *DSM 16992, could not be identified neither by BLAST searches, nor by geminivirus-specific pattern searches (see Methods). Since reasonable sequence conservation is a prerequisite for robust phylogenetic analysis, we did not incorporate RCR Rep sequences from other origins into our dataset.

**Figure 2:**
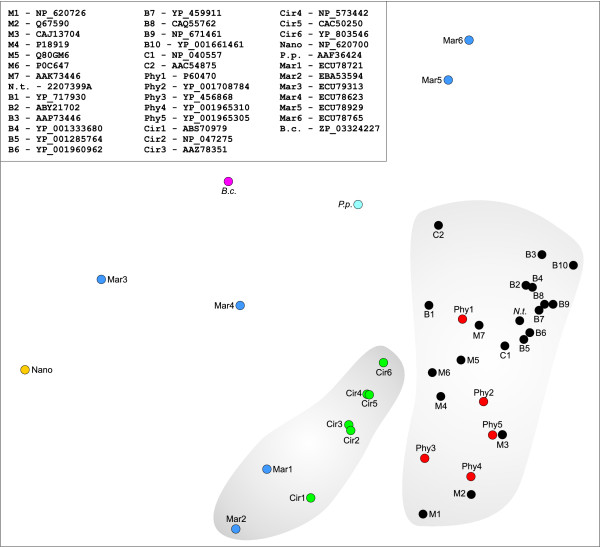
**Complete linkage clustering analysis of RCR Rep proteins**. Pairwise distance matrix for 40 Rep proteins was calculated using MEGA4 [51] and used for the clustering analysis. Distances between individual data points (colored circles) are proportional to the number of amino acid substitutions per site between sequences. GenBank accession number of each protein is indicated in the upper-left corner of the Figure. Abbreviations: B, begomovirus (black circle); C, curtovirus (black circle); M, mastrevirus (black circle); N.t., *Nicotiana tabacum *(black circle); phy, phytoplasmal plasmid (red circle); P.p., *Porphyra pulchra *plasmid (light blue circle); B.c., *Bifidobacterium catenulatum *DSM 16992 (magenta circle); Cir, circovirus (green circle); Nano, nanovirus (yellow circle); Mar, marine metagenome (blue circle).

The 40 sequences were aligned. The alignment was manually verified and edited [see Additional file 1]. A pairwise distance matrix was calculated from the alignment and used in the complete linkage clustering analysis (see Methods for details). All geminiviral Reps formed a single cluster (Fig. 2). Interestingly, Reps of phytoplasmal plasmids were found to be an integral part of the geminiviral cluster with individual data points dispersed within the cluster. Circoviral sequences clustered with two marine metagenomic sequences obtained during the Global Ocean Sampling Expedition. The rest of the sequences did not form clusters that would contain more than one sequence. The most divergent of the 40 sequences was Rep of a nanovirus [GenBank:NP_620700]. The pairwise distances between the nanoviral and other Reps were considerably larger than distances between any other pair of sequences (data not shown). Examination of the sequence alignment [see Additional file 1] revealed that the Rep protein of the nanovirus lacks the Walker B motif (DD) at the equivalent position in other Rep proteins. Furthermore, Walker A motif in nanovirus Rep (GxxGxxGKS), which was confirmed to be functional and essential for replication, differed from the canonical P-loop sequence (GxxxxGKT/S [11]). Therefore, nanoviral Rep was chosen as an outgroup in the following phylogenetic analyses.

**Figure 3:**
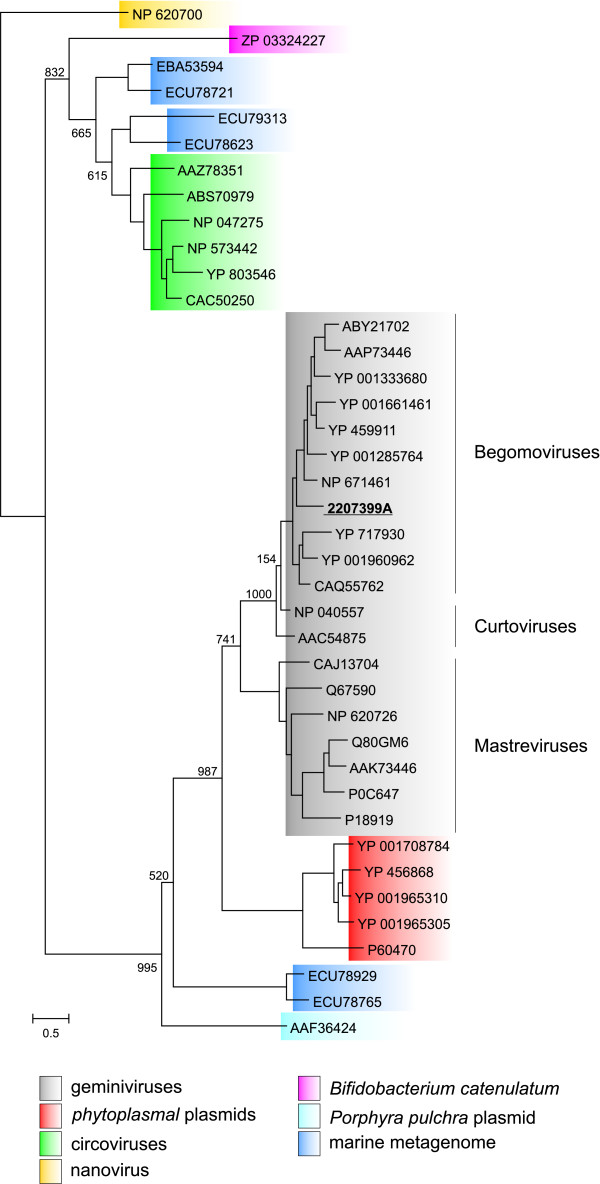
**Phylogenetic tree of the RCR Rep proteins**. Maximum likelihood tree was constructed using the PhyML program [23], with the WAG evolutionary model [50] with gamma distribution of rates between sites (four categories, alpha estimated by PhyML). Sequence alignment was constructed using CLUSTALW [49]. The nanovirus Rep was chosen as an outgroup to root the tree (see the main text for the outgroup selection). Numbers at the relevant branch-points represent bootstrap values (1000 replicates). Geminivirus-derived Rep of *Nicotiana tabacum *is underlined. The scale bar represents the number of amino acid substitutions per site.

Maximum-likelihood (ML) and Bayesian trees were inferred using PhyML [23] and MrBayes [24], respectively. The ML tree is shown in Fig. 3. All geminiviruses (including geminivirus-derived Rep from *N*. *tabacum *[18]) form a monophyletic group. The geminivirus clade, however, is divided into two clearly defined subgroups. One subgroup contains begomoviral and curtoviral sequences, where curtoviral sequences are at the base of the subclade. The second subgroup contains only mastreviral Rep sequences (Fig. 3). Interestingly, geminiviral Reps share a most recent common ancestor with plasmids of phytoplasmas and not with other ssDNA viruses, implying a separate origin for cicoviruses and possibly nanoviruses (see also below). Topology of the tree calculated using Bayesian inference was generally similar to that of the ML tree, predicting a more recent common ancestor for Reps from phytoplasmal plasmids and geminiviruses (compare Fig. 3 and Additional file 2. There were, however, slight differences in the branching within the geminiviral clade when compared to the ML tree. Position of the Rep from *B*. *catenulatum *DSM 16992 on the ML tree was different from that on the Bayesian tree.

When Rep proteins of phytoplasmal plasmids were searched for homologues using PSI-BLAST [25] against bacterial and viral databases at NCBI, only Rep protein sequences of other phytoplasmal plasmids or geminiviruses were identified with significant scores. This suggests that other bacterial RCR Rep proteins share much less similarity with phytoplasmal Reps than those of geminiviruses. Indeed, sequences of bacterial plasmid Reps identified using pattern searches by Koonin and Ilyina (1992) share only three of the five motifs characteristic to geminiviral Reps [15,17]. Also, there is no significant sequence similarity, other than the three shared motifs, between RCR Reps of bacterial plasmids (other than phytoplasmal plasmids) and geminiviruses. For example, BLAST searches against geminiviral protein sequences at NCBI using as seeds Rep sequences of plasmids pMV158 [GenBank:YP_001586272] and pUB110 [GenBank:CAA27141], the two plasmids whose Reps were found to be the closest to geminiviral Reps [17], returned no positive hits. Our analysis identifies Reps of phytoplasmal plasmids as the most similar sequences to geminiviral Reps from currently available public protein sequence databases. This observation suggests that geminiviral Reps share a more recent common ancestor with phytoplasmal plasmids than they do with other viral or plasmid RCR Reps.

Interestingly, phytoplasmas and geminiviruses are both obligate parasites occupying a common ecological niche – phloem tissue of plants, which consists of parenchyma cells, sieve-tube cells, and companion cells. Phytoplasmas have been observed in companion cells and phloem parenchyma cells as well as in sieve elements [21]. The same types of cells were shown to contain geminiviral DNA when *Nicotiana benthamiana *and *Lycopersicon esculentum *were infected with *Tomato yellow leaf curl Sardinia virus *and/or *Tomato yellow leaf curl virus *[26]. It should be noted, however, that not all geminiviruses are phloem-limited [27]. Furthermore, both geminiviruses and phytoplasmas share at least one common insect vector (leafhoppers) that is essential for transmission between plants [21,27]. It is conceivable that extrachromosomal replicons of phytoplasmas evolved by acquisition of the capsid-coding gene to give rise to geminiviruses.

**Figure 4:**
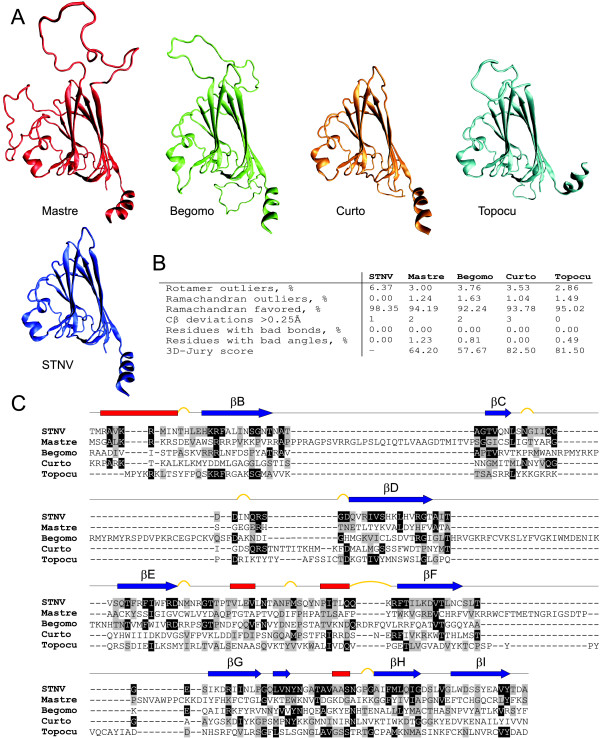
**Structural modeling of the geminiviral capsid proteins (CPs)**. (A) Pseudo-atomic models of the CPs of *Panicum streak virus *(Mastre; red), *Mesta yellow vein mosaic virus *(Begomo; green), *Horseradish curly top virus *(Curto; orange) and *Tomato pseudo-curly top virus *(Topocu; cyan) are compared to the atomic model of the CP of *Satellite tobacco necrosis virus *(STNV; blue; [PDB:2buk]). (B) Comparison of the stereochemical quality of the STNV CP X-ray structure to that of the pseudo-atomic models of geminiviral CPs. (C) Structure-based alignment of geminiviral CP sequences to the corresponding protein sequence of STNV. Residues that are identical or similar in the STNV CP and in at least one geminiviral sequence are boxed in black or gray, respectively. The secondary structure determined from the X-ray structure of STNV CP [PDB:2buk] is shown above the alignment with α helices, β strands, and turns represented by red rectangles, blue arrows, and yellow bulges, respectively. The nomenclature for the secondary structure elements (βB-βI) is also indicated [28].

In order to test this possibility, we focused on the capsid protein (CP) of geminiviruses. BLAST searches [25] against viral protein database at NCBI using CP sequences of geminiviruses as seeds revealed no possible homologues from viral families other than *Geminiviridae*. Since tertiary structure of the protein is usually more conserved than the primary one, structural comparisons of viral CPs have been previously proven to be useful by revealing connections between viral families that cannot be deduced from the sequence analysis alone [6,28]. Unfortunately, high resolution X-ray data on CP of geminiviruses is not available. We therefore approached structure prediction of CPs from four geminiviruses representing each of the four genera in the family *Geminiviridae*. Protein sequences of Panicum streak virus ([Swiss-Prot:Q00323];*Mastrevirus*), *Mesta yellow vein mosaic virus *([GenBank:]; *Begomovirus*), *Horseradish curly top virus *([GenBank:]; *Curtovirus*) and *Tomato pseudo-curly top virus *([GenBank:]; *Topocuvirus*) were downloaded from the NCBI protein database and submitted to the Structure Prediction MetaServer [29]. There are currently 231 icosahedral virus structures solved by X-ray crystallography and deposited in the Protein Data Bank (PDB). These structures are from bacterial, archaeal and eukaryotic viruses that belong to 29 different viral familes [30]. Out of all these icosahedral virus structures CP of *Satellite tobacco necrosis virus *(STNV) was found to be the only suitable template for structural modeling with significant scores for all four geminiviral CPs. In order to further corroborate this prediction we constructed 3D models of the four geminiviral CPs (Fig. 4A) and tested the stereochemical quality, along with the X-ray structure of the STNV CP (see Methods for details). Comparison of the obtained results (Fig. 4B) supported the reliability of the models indicating that CPs of geminiviruses have a potential to adopt the same fold as the CP of STNV – an eight stranded (βB-βI) β-barrel fold (with two sheets BIDG and CHEF) also known as the viral jelly-roll [28,31]. This observation leads to an intriguing conclusion that structurally similar viruses may employ different nucleic acids (RNA versus DNA) as their genetic material.

Next, we superimposed the structural models of the STNV and geminiviral CPs and extracted the structure-based sequence alignment (Fig. 4C). Of the 184 STNV CP amino acid residues for which structural information is available [PDB:2buk], 69.1% had corresponding amino acids in at least one of the four geminiviral CP sequences (75 identical and 52 similar residues) (Fig. 4C). Given the fact that all geminiviral CPs are true homologues, our observation indicates that STNV and geminiviral CPs share not only tertiary but also significantly similar primary structures which further justifies the suggested relationship between these viral CPs. It is obvious from Fig. 4 that secondary structure elements are well conserved and that insertions in the loop regions between β-sheets account for the larger size of geminiviral CPs. The most prominent insertions are observed in the CP of mastrevirus (between βB and βC, and between βF and βG) and begomovirus (between βC and βD, and between βD and βE). The βD/βE loop was identified as essential for controlling whitefly transmission of begomoviruses [32], whereas the βF/βG loop was proposed to be required for leafhopper transmission [33].

It is notable that the eight stranded β-barrel fold is characteristic to all icosahedral ssRNA plant and animal viruses [28] as well as to ssDNA viruses of the *Microviridae *and *Parvoviridae *families [34]. Previously, twinned particles of two geminiviruses, *Maize streak virus *(MSV; *Mastrevirus*) and *African cassava mosaic virus *(ACMV; *Begomovirus*), were resolved using electron cryo-microscopy (cryo-EM) and image reconstruction techniques to 25 Å [35] and 16–19 Å [33] resolution, respectively. In both studies the CP of STNV was also found to be the best template for structural modeling of the geminiviral CPs. Successful fitting of the pseudo-atomic model of MSV CP into the cryo-EM density map [35] strongly corroborates the prediction that CPs of STNV and geminiviruses share the same fold.

All these observations suggest a possible scenario for the origin of geminiviruses. Phylogenetic and clustering analyses of the geminiviral Rep proteins (Figs. 2, 3) indicate that they share a more recent common ancestor with Reps of plasmids from phytoplasmas rather than from other bacteria or viruses. There are two possible ways to explain this relationship. One is that a phytoplasmal cell, while being inside the plant cell, internalized the genome of a geminivirus-like agent, replication and partitioning of which was subsequently stabilized along with the loss of a CP-coding gene. The other possibility is that phytoplasmal plasmids released upon lysis of the bacterial cell in the cytoplasm of the host plant cell were able to obtain a capsid-coding gene from an unknown plant virus. The former possibility seems unlikely since some geminiviruses not only maintained features of prokaryotic replicons, such as typical bacterial promoter sequences [36], but what is more surprising, are in some instances still able to replicate their DNA in bacterial cells [37,38]. We were unable to identify any other proteins in addition to RCR Reps common to both, phytoplasmal plasmids and geminiviruses. However, this is not surprising, since protein content required for successful persistence inside bacterial (for plasmids) and plant (for geminiviruses) cells is likely to be different. Furthermore, the capsid volume is a limiting factor dictating the amount of genetic information that can be packaged. So, there is a strong pressure on the genome content of viruses with small capsids leading to the loss of genetic information unnecessary for virus propagation.

What virus might be a donor of a capsid-coding gene to the escaping phytoplasmal plasmid? The vast majority of plant viruses have RNA genomes. Modeling of the geminiviral CP suggests that it folds into the eight-stranded β-barrel (Fig. 4A), a fold common to all isometric ssRNA plant viruses. Notably, STNV encodes a single protein, a capsid protein, which was found to be the closest non-geminiviral relative of the geminiviral CP out of the 231 icosahedral virus capsid proteins whose X-ray structures are currently available at the PDB [30]. STNV possesses the simplest capsid formed from 60 subunits of the CP arranged into *T *= 1 icosahedral lattice [31]. Pentamers of the CP are the building blocks of the STNV particles [39]. The same is true for geminiviruses [34]. Geminivirus virions are composed of two incomplete icosahedra (110 copies of CP in MSV) that are joined together [35] (Fig. 1A). Such virion architecture is unique to geminiviruses and is not observed in any other currently known viruses. While the interior volume of the isometric particles is sufficient to pack 1,239 bp of the STNV genome, it is unable to accommodate the larger (2.5 – 3.0 kb [12]) genome of geminiviruses. Interestingly, it was found that the CP of geminiviruses produces not only twinned wild-type capsids but also isometric and even capsids formed of three incomplete icosahedra (Fig. 1) [40-42]. The valency of the capsid apparently correlates with the length of the packed nucleic acid. It has been shown that noninfectious isometric *T *= 1 MSV particles contain subgenomic MSV DNA fragments from about 0.2 kb to nearly half of the wild-type genome [40]. Such heterogeneity in particle size and production of noninfectious particles *per se *might be seen as an indication of ongoing optimization and adaptation of the CP, which was originally utilized to form smaller (isometric) particles, to build larger capsids. Taking into account the high nucleotide substitution rate in geminiviruses, which is similar to that of RNA viruses [43], the sequence conservation between STNV and geminiviral CPs as well as between phytoplasmal plasmid and geminiviral Reps is striking. It is possible that the emergence of the ancestor geminivirus from a phytoplasmal plasmid and an RNA virus occurred relatively recently on the evolutionary timescale. Although less likely, the possibility of the convergent evolution cannot be ruled out either.

An alternative hypothesis for the origin of geminiviruses is that they are descendants of as yet undiscovered ssDNA viruses with geminiviral-like Reps that have acquired their CP-coding genes either from an RNA or DNA virus by horizontal gene transfer. Indeed, recent metagenomic analysis of samples from a rice paddy soil unveiled the presence of putatively viral replicons with geminivirus/phytoplasma-like Reps but not other geminiviral genes [44]. Unfortunately, metagenomic studies do not provide any information on the origin of the amplified replicons, making it impossible to know with certainty that the amplified DNA does not belong to geminiviruses or plasmids. Therefore, there is currently no evidence to support the hypothesis predicting the existence of a virus that would be a missing link between geminiviruses and other ssDNA viruses.

If geminiviruses originated from phytoplasmal plasmids, is it possible that similar transitions happened several times to give rise to different viral families? As mentioned above, RCR Rep of the *Bifidobacterium pseudocatenulatum *plasmid p4M [GenBank:AAM00235] was previously shown to be more similar to Reps of various circoviruses than it is to Reps from other bacterial plasmids and viruses [19]. It is therefore tempting to speculate that circoviruses might also be direct descendants of bacterial plasmids.

## Conclusion

Phylogenetic as well as complete linkage clustering analysis of RCR Rep proteins from geminiviruses suggests their evolutionary relationship with Rep proteins of phytoplasmal plasmids, while structural modeling of the geminiviral CP points to a connection between geminiviruses and icosahedral ssRNA viruses. We suggest a scenario for the origin of geminiviruses in which acquisition of the capsid protein-coding gene from an ssRNA plant virus by phytoplasmal plasmid gave rise to the ancestor of geminiviruses. This scenario involves two assumptions. First, there was a coinfection of the same plant cell by a phytoplasma and an ssRNA virus. Indeed, such a coinfection has been previously observed. Sugarcane phloem was found to frequently contain both phytoplasmas and Sugarcane yellow leaf viruses (an icosahedral ssRNA virus) [45,46]. The second assumption is that recombination occurred between the RNA genome of a virus and the DNA molecule of a plasmid. Although recombination between RNA and DNA viruses is not common, there is evidence pointing to the possibility of such gene exchange in the viral world [47,48]. The scenario proposed here implies that geminiviruses emerged in plant cells through introduction of a structural element (capsid-coding gene) of a plant virus into a plasmid liberated from a plant infecting bacterium. Although this plasmid-to-virus transition does not satisfy the requirements of *de novo *virogenesis, since a preexisting viral building block was utilized for virion formation, it nevertheless accounts for the emergence of a novel virus family, the *Geminiviridae*. Consequently, the borderline between the two selfish genetic elements – viruses and plasmids – becomes transparent.

## Methods

### Data collection and phylogenetic analysis

Koonin and Ilyina (1992) found that geminiviral rolling-circle replication (RCR) initiation proteins (Rep) are related to certain bacterial Reps [17]. In order to obtain a dataset for phylogenetic analysis of geminiviral Reps we set out to get all bacterial RCR Reps from the nonredundant protein database at NCBI using PSI-BLAST searches (BLOSUM62 matrix, 0.05 as an E-value cutoff) [25]. Surprisingly, only RCR Reps from phytoplasmal plasmids were identified using this approach. To extend the dataset, we carried out an alternative approach, pattern matching. Rolling circle replication proteins of geminiviruses contain five conserved motifs that are essential for the activity [13-16]. Based on this knowledge, an exact geminivirus-specific sequence pattern, encompassing all the five conserved motifs, was generated: F(T [LI]/[LM]T) [YN]X(1,100)HX [HQ]X(1,100)YXXKX(50,200)GXXXXGK [ST]X(1,100)DD. The residues shown in square brackets are alternatives; X – any amino acid; numbers in parentheses denote the allowed distance between corresponding motifs; slash sign indicates alternation of the dipeptides in the second and third positions in the pattern. The non-redundant protein sequences and environmental protein sequences from BLAST database were downloaded (07.02.2009) from NCBI FTP site and searched for sequences exactly matching the derived pattern without paying attention to the sequences surrounding the conserved motifs (as long as their length falls in the range specified in the pattern). Using this approach sequences missed by BLAST searches are expected to be found. 1072 protein sequences were initially extracted. In order to avoid redundancy, the original dataset was subsequently filtered to leave only sequences with less than 70% identity. As a result, a dataset containing 43 protein sequences was obtained. Of these two sequences were false-positive – a 799 amino acid-long hypothetical protein [GenBank:XP_001614627] from *Plasmodium vivax *SaI-1 and a 440 amino acid-long hypothetical TrmE domain protein GOS_1133298 [GenBank:EDE42344] from marine metagenome project, which were not included in the further analysis. The resultant dataset (41 sequences) was used to create a multiple sequence alignment using CLUSTALW [49]. One geminiviral sequence [GenBank:ABD67440] was found to be considerably longer (469 aa) than the rest of the sequences. The protein was found to be a fusion of RCR Rep and geminiviral transcriptional activator AC2 and was therefore removed from the alignment. The 40 sequences were realigned and following manual examination and editing the subsequent alignment [see Additional file 1] was utilized for phylogenetic analysis. Maximum likelihood analysis was carried out by using PhyML v2.4.4 [23], with a WAG [50] model of amino acid substitution, including a gamma law with 4 categories to take into account differences in evolutionary rates at sites, and an estimated proportion of invariable sites. The robustness of the tree was assessed by bootstrap analysis (1,000 replicates). Bayesian phylogenetic tree was constructed using MrBayes [24] with a mixed model of amino-acid substitution and a Gamma-law (eight discrete classes). MrBayes was run with four chains for 2.1 × 10^6 ^generations and trees were sampled every 100 generations. To construct the consensus tree, the first 25% of the trees were discarded as "burnin".

### Complete linkage clustering analysis

Multiple sequence alignment [see Additional file 1] was used to calculate the pairwise distance matrix with MEGA4 [51]. Analyses were conducted using the Poisson correction method. All positions containing gaps and missing data were eliminated from the dataset (Complete deletion option). There were a total of 178 positions in the final dataset. The calculated pairwise distances were used to perform complete linkage clustering analysis, where the distance between two clusters is defined as the distance between the two farthest objects in the two clusters. At each round the clusters are examined and split to two clusters according to the longest distance. The members of the clusters were then grouped within the new cluster that has a shorter distance. The clustering was run until all sequences formed their own clusters.

### Structural modeling

BioInfoBank MetaServer [29] was used for prediction of the tertiary structures. The structure of STNV capsid protein (CP) [31] was determined to be the best template for structural modeling with significance scores ranging from 57.67 – 82.50; scores above 50 are assumed to be significant and correspond to a prediction accuracy of above 90% [29]. The sequences of the geminiviral CPs were individually aligned with the corresponding protein sequence of STNV using version 9.2 of the MODELLER program [52]. Align2d algorithm of the MODELLER program is different from standard sequence-sequence alignment methods because it takes into account structural information from the template when constructing an alignment. This task is achieved through a variable gap penalty function that tends to place gaps in solvent exposed and curved regions, outside secondary structure segments, and between two positions that are close in space. The resulting alignments were utilized to build the three-dimensional models of the four geminiviral CPs using the MODELLER. Ten variants of each CP were generated and one of them was chosen on the basis of having the best stereochemical quality, which was validated using MolProbity [53]. The structural superpositioning of the models with the X-ray structure of the STNV CP was performed using the STAMP algorithm [54], and the results were visualized with the VMD program [55].

## Authors' contributions

MK conceived the project, collected, analyzed and interpreted the data, and drafted the manuscript. JJR collected and analyzed the data, and revised the manuscript. DHB interpreted the data and revised the manuscript. All authors read and approved the final manuscript.

## Supplementary Material

Additional file 1 **Multiple sequence alignment of 40 RCR Rep proteins**. Figure shows a multiple sequence alignment which has been used to calculate the phylogenetic trees. Click here for file

Additional file 2 **Bayesian consensus tree of the RCR Rep proteins**. Figure shows the Bayesian consensus tree which has been calculated using the same dataset as for the Maximum likelihood tree shown in Figure 3. Click here for file
