# Supplementary material for: Geminiviruses: a tale of a plasmid becoming a virus
Source: BMC Evol Biol. 2009 May 21;9:112. doi: 10.1186/1471-2148-9-112 (PMC2702318; doi:10.1186/1471-2148-9-112)
Supplement: Additional file 1 — Multiple sequence alignment of 40 RCR Rep proteins. Figure shows a multiple sequence alignment which has been used to calculate the phylogenetic trees. [file 1471-2148-9-112-S1.pdf]

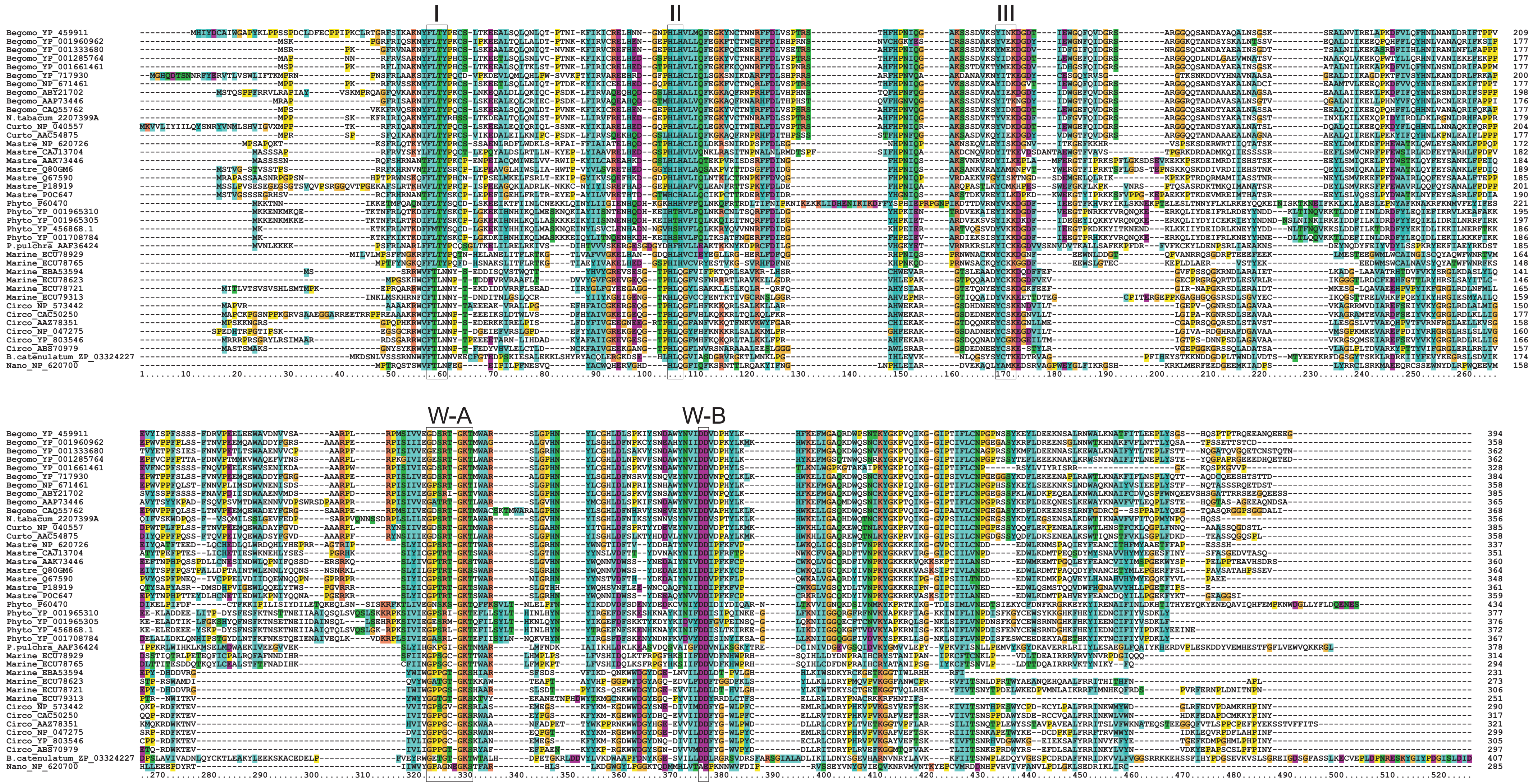

Figure S1. Multiple sequence alignment of 40 RCR Rep proteins. The five motifs conserved in geminiviral Reps are boxed. W-A, Walker A motif; W-B, Walker B motif. The alignment was constructed using CLUSTALW [1] and then edited manually.

Reference:

1. Thompson JD, Higgins DG, Gibson TJ: **CLUSTAL W: improving the sensitivity of progressive multiple sequence alignment through sequence weighting, position-specific gap penalties and weight matrix choice**. *Nucleic Acids Res*. 1994 **22**:4673–4680.
