# Supplementary material for: Geminiviruses: a tale of a plasmid becoming a virus
Source: BMC Evol Biol. 2009 May 21;9:112. doi: 10.1186/1471-2148-9-112 (PMC2702318; doi:10.1186/1471-2148-9-112)
Supplement: Additional file 2 — Bayesian consensus tree of the RCR Rep proteins. Figure shows the Bayesian consensus tree which has been calculated using the same dataset as for the Maximum likelihood tree shown in Figure 3. [file 1471-2148-9-112-S2.pdf]

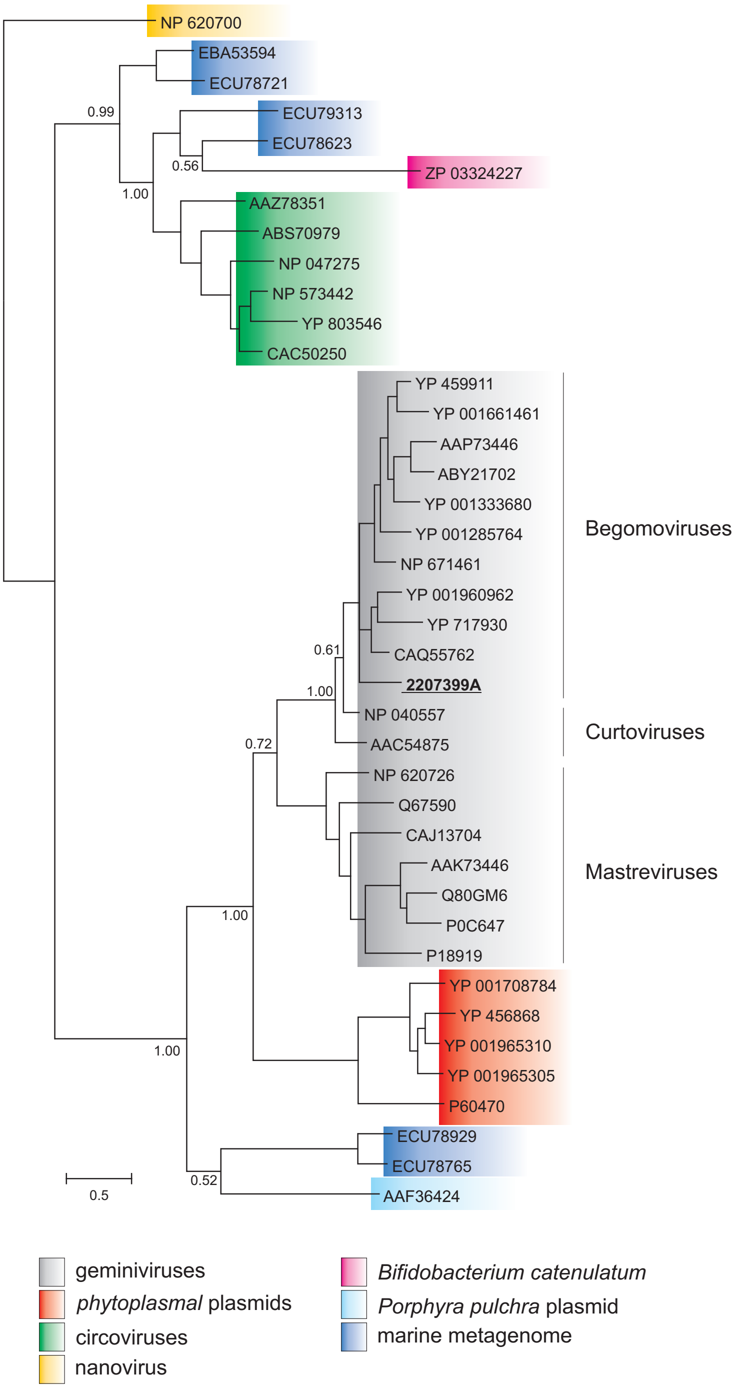

Figure S2. Bayesian consensus tree of the RCR Rep proteins. The nanovirus Rep was chosen as an outgroup to root the tree (see the main text for the outgroup selection). Numbers at the relevant branch-points represent posterior probabilities. Geminivirus-derived Rep of *Nicotiana tabacum* is underlined. The scale bar represents the number of amino acid substitutions per site.
